# Editorial: Immune-related biomarkers in skin and breast cancer: innovations in immunological diagnostics and therapies

**DOI:** 10.3389/fimmu.2026.1901169

**Published:** 2026-06-17

**Authors:** Domenico Mallardo, Margaret Ottaviano, Giuseppe Palmieri, Maria Teresa Melucci

**Affiliations:** 1 Melanoma, Cancer Immunotherapy and Development Therapeutics Unit, Istituto Nazionale Tumori, Istituto di Ricovero e Cura a Carattere Scientifico (IRCCS) 'Fondazione G. Pascale', Naples, Italy; 2 Immuno-Oncology & Targeted Cancer Biotherapies, University of Sassari, Unit of Cancer Genetics, Istituto di Ricerca Genetica e Biomedica - Consiglio Nazionale delle Ricerche (IRGB-CNR), Sassari, Italy; 3 Breast Surgery with Intraoperative Radiotherapy (IORT), Istituto Nazionale Tumori-IRCCS - Fondazione “G. Pascale”, Naples, Italy

**Keywords:** breast cancer, cytokines, immune-related biomarkers, immunology, immunotherapies, skin cancer, tumor microenvironment

## Introduction

Cutaneous melanoma remains a reference model for cancer immunology. Its high mutational burden, metastatic potential and capacity to remodel the tumor-immune microenvironment explain both its clinical aggressiveness and its sensitivity to immune checkpoint blockade (1, 2). CTLA-4 and PD-1/PD-L1 inhibitors have transformed advanced melanoma, producing durable survival in a substantial subgroup of patients, while neoadjuvant nivolumab plus ipilimumab has extended this immunological logic to resectable stage III disease (3–7). Yet resistance and immune-related toxicity remain decisive limitations. PD-L1 expression, tumor mutational burden, interferon-gamma-related programs, TILs, antigen presentation, circulating immune subsets, cytokines, proteomic signatures, drug exposure, microbiome and metabolic features have each been linked to outcome, but none captures the full biology of response (8–17).

Non-melanoma skin cancers and breast cancer underscore the same problem from different biological angles. Merkel cell carcinoma (MCC) and cutaneous squamous cell carcinoma (cSCC) are immunotherapy-responsive tumors, with durable activity of PD-1/PD-L1 blockade, high pathological response rates after neoadjuvant cemiplimab in cSCC, and emerging evidence that systemic inflammation may shape outcome (18–23). Breast cancer is more heterogeneous: triple-negative and HER2-positive tumors are usually more immune enriched than hormone receptor-positive disease; pathological complete response is prognostically informative; checkpoint inhibition improves outcomes in selected patients (24–31). Across these settings, biomarkers must therefore move beyond association and become decision tools for treatment selection, escalation or de-escalation, early response monitoring, toxicity prediction and treatment discontinuation.

**Table 1** summarizes the biomarker axes and translational implications of the 20 Research Topic contributions.

**Table 1:** Overview of immune-related biomarkers, biological axes, and translational implications across the breast and skin cancer studies included in this Research Topic.

Tumor setting	Study focus	Biomarker/biological axis	Main translational implication
Breast cancer	SUMOylation-related molecular patterns (He et al.)	NR3C2, CDCA8, AURKA, PLK1; SUMOylation subtypes	Links post-translational regulation, proliferative stress, DNA repair and immune contexture.
Breast cancer	Hedgehog pathway-related genes (Wang and Wang)	Hedgehog-related score	Connects developmental signaling, stromal remodeling, immune infiltration and prognosis.
Breast cancer	Immune inhibitory receptor biology (Shi et al.)	PILRA	Expands immune-regulatory stratification beyond PD-1/PD-L1.
Breast cancer	Cellular-therapy target discovery (Xu et al.)	GRM4	Identifies a candidate antigen for future CAR- or antibody-based strategies.
Breast cancer	Mitophagy and mitochondrial quality control (Zhao et al.)	Mitophagy signature; PBK, NEK2	Links mitochondrial fitness, proliferation, metabolism and immune regulation.
Breast cancer	Systemic inflammation and neoadjuvant response (Wu et al.)	NLR, MLR, PLR, systemic immune-inflammation index	Provides accessible blood biomarkers associated with pCR and disease-free survival.
Breast cancer	Plasma cytokine profiling and treatment response (Vela-Ruiz et al.)	MIP-1beta, IL-9, GRO-alpha, TNF-beta, FGF-BASIC, PDGF-BB, SDF-1, IL-12 p40, IL-8	Supports cytokine-based stratification of subtype and treatment response in stage II-III disease.
Breast cancer	Multimodal prediction of neoadjuvant response (Yan et al.)	Longitudinal DCE-MRI, TILs, inflammatory indices	Integrates imaging, local immunity and systemic inflammation for dynamic response prediction.
Breast cancer	Radiomics and proteomics (Li et al.)	Ultrasound radiomics plus plasma proteomics	Combines non-invasive imaging and molecular information for diagnostic precision.
Breast cancer	Genomic-pathological artificial intelligence (Bilal et al.)	BCDNN integrating genomic and pathological data	Links tissue morphology and molecular features in computational diagnosis.
Skin cancer	Multidisciplinary care and flap salvage (Chen et al.)	Postoperative necrotic flap management	Highlights clinical complexity after aggressive skin tumor surgery.
Cutaneous melanoma	Integrated stress response (Zhang et al.)	DTL, DTX3L, KCNMB1, NDRG1, GPX2, DERL3, MBTPS2	Links cellular stress adaptation with immune landscape and prognosis.
Cutaneous melanoma	Tryptophan metabolism (Zhao et al.)	HADHA, GOT2, STAT1, CAT, IDO1	Defines an immunometabolic axis with prognostic and immune relevance.
Cutaneous melanoma	Human endogenous retrovirus profiling (Fei et al.)	HERV expression patterns	Captures retroviral and epigenetic sources of immunogenicity and heterogeneity.
Cutaneous melanoma	Extracellular vesicles (Christodoulides et al.)	EV cargo and tumor-host communication	Supports liquid-biopsy approaches to immune evasion and resistance.
Skin cancer	Transcriptomic technologies (Ma et al.)	Bulk, single-cell and spatial transcriptomics	Adds cellular and spatial resolution to immune biomarker discovery.
Skin cancer	Immune-related adverse events (Flatt et al.)	IL-1RA, CXCL13, IL-7 cytokine score	Suggests circulating cytokines may predict toxicity and therapeutic index.
Merkel cell carcinoma	Clinical prognostic stratification (Brenner et al.)	Head and neck location	Identifies anatomical site as a clinically accessible prognostic variable.
Cutaneous squamous cell carcinoma	Treatment duration and complete response (Fung and Samlowski)	Complete response; cemiplimab discontinuation; duration	Supports response depth as a functional biomarker for treatment duration.
Skin cancer	Explainable artificial intelligence (Fiaz et al.)	Deep-learning segmentation and multiclass classification	Complements clinical diagnosis with future molecular and immune integration.

## Breast cancer: from molecular association to clinically useful immune stratification

The breast cancer contributions show a field moving from single markers to layered biological states. He et al. place SUMOylation within proliferation, DNA repair and immune-contexture remodeling, suggesting that post-translational regulation may indirectly shape tumor antigenicity, interferon signaling and immune escape. Wang and Wang connect Hedgehog signaling with stromal architecture, immune infiltration and therapeutic vulnerability, a relevant framework for tumors in which developmental plasticity and immune exclusion coexist. Shi et al. identify PILRA as a candidate inhibitory immune axis, extending breast cancer checkpoint biology beyond PD-1/PD-L1 and pointing toward myeloid or inhibitory immune states that may be missed by conventional markers. In parallel, Xu et al. propose GRM4 as a tumor-selective antigen for future cellular or antibody-based strategies, addressing the central obstacle of target specificity in solid-tumor immunotherapy. Read alongside established genomic classifiers and earlier studies linking DNA repair and metastatic biology to outcome (32–36), these papers emphasize a critical principle: biomarkers become clinically useful only when they are tied to an actionable decision, not merely to prognosis. Another strength of these molecular studies is that they connect immune biomarkers with canonical cancer biology rather than treating immunity as a separate compartment. SUMOylation, Hedgehog signaling, inhibitory receptor biology and antigen discovery are not competing explanations; they describe different routes through which tumor cells, stroma and immune cells co-evolve under therapeutic pressure. This framing is particularly useful in breast cancer, where receptor-defined subtypes remain clinically essential but insufficient to explain immune responsiveness.

Metabolic and inflammatory programs add a second, more dynamic layer. Zhao et al. link mitophagy-related genes, including PBK and NEK2, with prognosis, immune landscapes and proliferative fitness, connecting mitochondrial quality control with tumor adaptation and immune regulation. This is biologically important because mitochondrial stress can influence innate immune sensing, antigen presentation and cell survival under therapy. Wu et al. show that systemic inflammatory indices, particularly the systemic immune-inflammation index, are associated with pathological complete response and disease-free survival in HER2-positive disease. The prospective Peruvian cohort study further refines this concept in 88 women with stage II-III breast cancer: MIP-1beta, IL-9, GRO-alpha and TNF-beta differed across molecular subtypes, lower FGF-BASIC was associated with pCR, and reduced FGF-BASIC, PDGF-BB and IL-12 p40 remained independently linked to clinical response (Vela-Ruiz et al.). Together, these studies suggest that blood-based inflammatory and cytokine markers should not be treated as crude surrogates, but as scalable readouts of host-tumor interaction that may complement tissue biomarkers in populations underrepresented in biomarker research.

The third layer is methodological integration. Yan et al. combine longitudinal DCE-MRI, TILs and peripheral inflammatory indices, treating neoadjuvant response as a temporal process rather than a baseline state. This distinction matters: baseline immune infiltration may describe potential, but early changes under treatment reveal biological behavior. Li et al. integrate radiomics and proteomics, while Bilal et al. combine genomic and pathological data through deep learning. These approaches are not interchangeable technical exercises. Imaging captures spatial heterogeneity and vascular-stromal structure, proteomics reflects tumor and host-response signals, and digital pathology can encode tissue architecture at scale. Their common challenge will be interpretability, reproducibility and prospective validation. Their common message is stronger: credible breast cancer immune stratification will require multimodal models that connect molecular biology, local immune contexture, systemic inflammation and clinical timing to decisions that can change treatment. A critical issue, however, is that these models should not be evaluated only by discrimination metrics. For editorial and clinical relevance, the key questions are whether a model changes the timing of surgery, identifies patients who can safely avoid additional therapy, or recognizes non-responders early enough to justify treatment adaptation. The most promising contribution of these breast cancer studies is therefore conceptual as well as technical: they shift biomarker research from static classification toward treatment-aware prediction. Their limitation is equally clear. Most signatures still require external validation, harmonized assays, and demonstration that their use improves outcomes rather than simply improves prediction. Future studies should therefore embed these tools in prospective neoadjuvant trials, where pCR, residual disease, toxicity and survival can be connected to pre-specified clinical actions.

## Skin cancer: biomarker expansion beyond PD-L1, TMB, and TILs

The skin cancer contributions span melanoma, Merkel cell carcinoma (MCC) and cutaneous squamous cell carcinoma (cSCC). Collectively, they show that immune-related biomarkers in cutaneous oncology are expanding beyond PD-L1 expression, tumor mutational burden and TILs. The report on salvage of a necrotic flap after malignant peripheral nerve sheath tumor excision also usefully anchors this biomarker discussion to surgical complexity, wound repair and multidisciplinary care, reminding us that immune-oncology biomarkers must ultimately operate in real clinical pathways, not in molecular abstraction (Chen et al.). This is particularly relevant in cutaneous oncology, where immune sensitivity is high but clinical trajectories remain heterogeneous across melanoma, MCC, cSCC and rarer tumors. The collection therefore supports a broader definition of biomarker: not only a molecule measured in tissue or blood, but any reproducible biological or clinical signal that clarifies immune competence, risk or therapeutic trajectory.

In melanoma, Zhang et al. identify an integrated stress-response model that links cellular adaptation with distinct immune landscapes, highlighting resistance as an active survival program rather than a passive absence of immunity. Zhao et al. define a tryptophan metabolism-related signature including IDO1, a pathway with strong immunosuppressive rationale across cancers (37, 38). This places immunometabolism at the center of melanoma stratification and provides a biological bridge to other tumors. Fei et al. add endogenous retrovirus profiling, suggesting that retroelement expression may capture epigenetic and transcriptional sources of tumor immunogenicity beyond mutation burden. Christodoulides et al. synthesize the extracellular vesicle field, supported by evidence linking melanoma exosomes, metastatic education and exosomal PD-L1 to immune escape and response (39, 40). Together, these studies move melanoma biomarkers from static tumor descriptors toward stress, metabolism, viral mimicry and liquid-biopsy dynamics.

Additional studies broaden the clinical perimeter. Ma et al. position bulk, single-cell and spatial transcriptomics as complementary tools for resolving immune states and tissue architecture; this is essential because immune infiltration is clinically meaningful only when cell state and spatial localization are understood. Flatt et al. propose a circulating cytokine score including IL-1RA, CXCL13 and IL-7 for early identification of patients at high risk of immune-related adverse events, bringing therapeutic index into biomarker development. Brenner et al. show that head and neck location carries adverse prognostic information in local and locally advanced Merkel cell carcinoma, reminding us that anatomical variables may reflect biology, lymphatic drainage and treatment complexity. In cutaneous squamous cell carcinoma, Fung and Samlowski report durable disease control after elective cemiplimab discontinuation in complete responders, a concept aligned with real-world data on treatment duration (41) and with the idea that depth of response may function as a clinical biomarker. The corrected deep-learning study adds diagnostic AI (Fiaz et al.). Thus, skin cancer biomarkers should integrate tumor adaptation, spatial biology, systemic immunity, toxicity, response depth and digital diagnostics. This breadth is important because melanoma and non-melanoma skin cancers do not fail immunotherapy through a single mechanism. Some tumors are poorly primed; others are metabolically suppressive, spatially excluded, myeloid-rich, transcriptionally plastic, or exposed to host inflammatory states that limit effective immunity. The studies collected here capture these layers from different angles. Stress-response and tryptophan signatures address tumor-intrinsic adaptation; HERVs and extracellular vesicles extend the discussion to non-mutational immunogenicity and liquid biopsy; transcriptomics restores cellular and spatial resolution; cytokine profiles address toxicity; and response duration introduces a clinical, treatment-dynamic biomarker. The unresolved challenge is integration. A useful melanoma or CSCC biomarker panel will likely need to combine tumor antigenicity, immune localization, systemic inflammation, toxicity risk and early treatment response in a format that remains simple enough for clinical use.

## Conclusions and perspectives

This Research Topic argues for a pragmatic shift in immune-related biomarker science. Focusing on breast and skin cancers, malignancies with so different immunophenotypes - in terms of both extracellular (immune infiltrate rates, tumor microenvironment status, etc.) and intracellular (quantitative and qualitative molecular alterations such as TMB and pathogenetic mutations, respectively) features, is precisely in the direction of overcoming the use of conventional markers for the assessment of immune-responsiveness or -resistance of tumors. In breast cancer, the most informative signals are likely to emerge from the integration of tumor-intrinsic programs, cytokine biology, inflammation, imaging and computational pathology. In skin cancer, biomarker development is converging on stress adaptation, immunometabolism, retroelement biology, extracellular vesicles, transcriptomic architecture, toxicity prediction and response durability. Across both fields, the goal is no longer to catalog plausible markers, but to build reproducible tools that can guide diagnosis, treatment selection, early adaptation, de-escalation and discontinuation. The next phase will depend on prospective validation, technical standardization and models that are interpretable enough to support clinical decisions. Importantly, this integrated view should not dilute clinical applicability. The field needs fewer isolated candidate markers and more decision-oriented signatures: markers that indicate when to intensify therapy, when to stop, when to monitor more closely and when to shift strategy. In this sense, the articles in this Research Topic provide a coherent roadmap: immune-related biomarkers in breast and skin cancer are becoming multidimensional, but their value will be measured by their capacity to alter patient management.
